# The Medical Library Association Guide to Data Management for Librarians

**DOI:** 10.5195/jmla.2017.270

**Published:** 2017-07-01

**Authors:** Mary A. Wickline

Big data, data science, data management, data curation—beyond buzz words, these iterations describe the recording, reuse, and long-term care of research data. The need for management and curation also indicates the usefulness and value of libraries and information science to a broad base of researchers, universities, and clinical scientists.

Lisa Federer, AHIP, of the National Institutes of Health (NIH), along with authors from the National Library of Medicine (NLM) and highly reputable universities, introduces readers to the theoretical and data life cycle aspects of data management and then offers practical examples from academic health sciences and hospital environments. Each chapter has footnotes and a bibliography, as well as pearls of wisdom and recommended reading. The book itself is indexed and logically organized. The theoretical section begins with a chapter from an NLM associate director, Valerie Florance, who maps out the landscape of data needs, organizational or grant mandates, existing datasets that are publicly available, and the recommendations of the NIH Data and Informatics Working Group, which focus on sharing data, supporting methodologies, training the workforce, and funding commitments.

Chris Eaker’s chapter on the impact of poor data management speaks to the reasons every institution and researcher should care deeply about good data management. Eaker begins by citing studies that found that 67% of article retractions were from scientific misconduct and that an inability to replicate studies costs $28 billion per year. The chapter illuminates “what can go wrong”—in planning, data collection, quality assurance, documentation, preservation, and analysis of data—with responses demonstrating specific tools and methods to show “what can be done differently” (pp. 14–23).

A dozen years ago, Philip Bourne sought to make the importance of data management clear when he talked about referencing the data and not just the previous analysis in scientific papers [1]. The amount of data now generated from one experiment makes additional analysis a driving tool for moving science forward. Federer makes that importance concrete with her example in chapter 6 of a computer programmer, using publicly available data, who successfully wrote code aimed at discovering harmful mutations in tumor suppressor genes (benign versus malignant tumor likelihood in BRCA1) through gene expression profiling—who that programmer is offers a delightfully surprising case for why publicly available data are so valuable (p. 69).

Access to data enables verification, replication, and multiple minds working on focused problem solving. Confirmation bias is human nature [2], and by its very nature, even scientists can initially be unaware of confirmation bias in their own work [3]. Whether by error or intention, the day will come when researchers’ nonresponse or reluctance to share data or to make data publicly available will cast their credibility in doubt. Science is a series of building blocks. If one researcher makes a mistake in analysis, subsequent papers that rely on that analysis without original data further the error instead of correcting the science.

Systematic data management allows researchers to dig deep, verify, and build further on a solid foundation that can be accurately assessed. Data management is about accessibility and additional analysis as well as the ability to respond to requests to “show me your data.” Many scientists’ data are in stacks of paper on physical desks, or data logs stored in metal filing cabinets, or even in files that cannot be found for reasons of naming, organization, or just the minutia of the data point not being searchable. All of this prior, typical disorganization supports the need for data management.

As data management is a somewhat new field, there are many recent books published on the topic, and librarians would do well to read widely. Robin Rice and John Southall’s *The Data Librarian’s Handbook* (Facet Publishing; 2016; ISBN: 978-1-78330-047-1) pitches the importance of attention to data for librarians, and Christine Borgman’s *Big Data, Little Data, No Data* (MIT Press; 2015; ISBN: 978-0-262028561) takes a deeper intellectual dive into the theoretical imperative. However, the *Medical Library Association Guide to Data Management—*with its wealth of knowledgeable, experienced chapter authors—hits the right note in balancing policies, theory, and practical considerations in an easy-to-read but thorough introduction to the topic.

The Medical Library Association Guides have consistently provided high-quality content that is well organized and indexed, and this book is no exception. The *Medical Library Association Guide to Data Management for Librarians* would be an excellent textbook for survey courses in data curation for health or biomedical library science students. Even more importantly, it is also a well-organized, useful reference for current librarians and information scientists who seek skills to support the direction that library science is leading and grant-funding agencies are demanding in publications. Data management is a natural fit for librarians—we have, after all, been managing information for hundreds of years. We must keep current, and Federer’s book is a welcome resource.
